# Reliability and construct validity of the stepping-forward affordance perception test for fall risk assessment in community-dwelling older adults

**DOI:** 10.1371/journal.pone.0225118

**Published:** 2019-11-20

**Authors:** Gabriela Almeida, Jorge Bravo, Hugo Folgado, Hugo Rosado, Felismina Mendes, Catarina Pereira

**Affiliations:** 1 Departamento de Desporto e Saúde, Escola de Ciências e Tecnologia, Universidade de Évora, Évora, Portugal; 2 Comprehensive Health Research Center, Lisboa, Portugal; 3 Escola Superior de Enfermagem S. João de Deus, Universidade de Évora, Évora, Portugal; University of Malaya, MALAYSIA

## Abstract

Thus far, few studies have examined the estimation and actual performance of locomotor ability in older adults. To our knowledge, there are no studies examining the relationship between stepping-forward estimation versus ability and fall occurrence. The aim of this study was to develop and assess the reliability and validity of a new test for fall risk assessment in community-dwelling older adults. In total, 347 participants (73.1 ± 6.2 years; 266 women) were assessed for their perception of maximum distance for the stepping-forward and action boundary. The test was developed following the existing literature and expert opinions. The task showed strong internal consistency. Intraclass correlation ranged from 0.99 to 1 for intrarater agreement and from 0.83 to 0.97 for interrater agreement. Multivariate binary regression analysis models revealed an area under the curve (AUC) of 0.665 (95% CI: 0.608–0.723) for fallers and 0.728 (95% CI: 0.655–0.797) for recurrent fallers. The stepping-forward affordance perception test (SF-APT) was demonstrated to be accurate, reliable and valid for fall risk assessment. The results showed that a large estimated stepping-forward associated with an underestimated absolute error works as a protective mechanism for fallers and recurrent fallers in community-dwelling older adults. SF-APT is safe, quick, easy to administer, well accepted and reproducible for application in community or clinical settings by either clinical or nonclinical care professionals.

## Introduction

Falls cause death, morbidity, dependence, and loss of quality life [1]. An accurate assessment of the risk of falling in older adults is essential to design proper interventions for those who are at risk of falling. Several studies have focused on identifying risk factors that are determinants of fall occurrence, such as environmental hazards, physical activity levels, physical fitness or cognition status [2–6]. However, the predictive and discriminative ability of these models and instruments to explain fall occurrence is generally low to moderate [7, 8], suggesting that there is a gap that traditional fall risk assessment instruments do not fill [9]. The assessment of affordance perception could be one of the key components considered in the current assessment of fall risk.

To successfully perform an action in the environment, each person needs to recognize their action boundaries. The possibilities for action are dependent on the fit between the environment and an individual’s action capabilities; that is, individuals need to be able to perceive what actions are possible within the limits of their capabilities [10]. This relation between perception and action is based on Gibson’s ecological framework [11, 12]. A central concept of his theory of perception and action is affordances, that is, opportunities for actions under a particular set of conditions and body characteristics [13]. Aging decline and alterations of functions and capabilities can contribute to an inaccurate perception of action boundaries and can lead to a perceptual misestimation, particularly in postural [14] and locomotor skills [15]. Any perceptual misestimation in locomotor skills in older adults can potentially lead to balance loss or accidental falls [16, 17]. Hence, what at an early stage of life was perceived as an affordance, in older ages, may not be. Therefore, aging-associated misperception of affordance perception can lead to a higher risk of falling in older adults, perhaps due to difficulties in actualizing the new limits for action [14] considering individual characteristics and perceptual attunement with the information.

Studies targeting perception-action capabilities under the ecological approach conducted on older adults have focused mainly on stair climbing [18], which represents a common everyday action. Since previous studies showed that falls occur during ordinary actions in daily life, such as walking [19], it is important to design tools measuring the perception of affordances for locomotor skills.

We hypothesized that the ability to perceive action boundary accurately for the stepping-forward skill may serve as an indicator of the risk of fall occurrence on community-dwelling older adults. Nonetheless, to the best of our knowledge, there are no valid tests to evaluate older adults’ perception of their maximum stepping-forward distance, particularly to assess their risk of being a faller or a recurrent faller. Therefore, we designed a test to assess the stepping-forward affordance perception using the locomotor task of stepping forward. The test design was motivated by Gibson’s ecological approach [11, 12], which underlies the potential actions afforded by the environment. The test’s protocol is within that of other experiments to study affordance perception in older adults, wherein participants were first asked to identify the perceived maximum performance, following an action boundary establishment [18]. Considering the above, the aim of the present study was to develop and assess the validity and reliability of the stepping-forward affordance perception test (SF-APT) for fall risk assessment in community-dwelling older adults.

## Material and methods

### Participants

Volunteers for this study (367 Portuguese community-dwelling older adults) were enrolled via pamphlets placed in community settings (health, recreational, sports, cultural and senior centers). The inclusion criteria were as follows: adults ≥65 years old with independent mobility, absence of fall occurrence due to the performance of hazardous and unusual tasks, and absence of cognitive impairment in accordance with the Portuguese version of the Mini-Mental State Examination [20, 21]. The sample size was estimated to be 271 by the online OpenEpi software (http://www.openepi.com/SampleSize/SSCohort.htm), keeping the confidence interval (CI) at 90% and level of significance at 5%. Eleven volunteers did not meet the criterion of absence of cognitive impairment, and 9 did not meet the criterion of absence of fall occurrence due to the performance of unusual and hazardous tasks. A total of 347 participants (266 women and 81 men) remained, aged 73.1 ±6.2 years, with 5.2 ±3.3 years of school attendance, a body mass index of 28.8 ±3.9 m/kg^2^, a body fat mass percentage of 37.6 ±8.9% and a body lean mass percentage of 26.69 ±4.6%. Of the 347 participants, 201 did not fall in the previous year, and 146 had falls at least once in the previous year, of which 62 had fallen more than once. Thirty participants (73.3 ± 5.83 years) participated in the intrarater reliability procedure, and 34 (75 ± 6.7 years) participated in the interrater reliability procedure. Written informed consent was obtained from all participants, and ethical approval was granted by the Universidade de Évora–Comissão de Ética para a Investigação Científica nas Áreas de Saúde Humana e Bem-Estar (reference number 16–012).

### Procedures

The SF-APT was inductively designed from a review of the literature in order to identify the conceptual frameworks related within the perception of affordances and falls and expert consulting. The task, goals, instructions, and measured variables that should be included in the test were outlined, and a refinement was performed based on expert opinion and against observed task performance, ensuring content validity.

#### The stepping-forward affordance perception test

SF-APT performance involves a first training attempt (trial) and a second measurable attempt (scoring). Both test trial and scoring tasks are performed on a uniform floor surface but in different locations. The test begins with the rater providing a verbal explanation followed by the trial, with no feedback.

**Fig 1:**
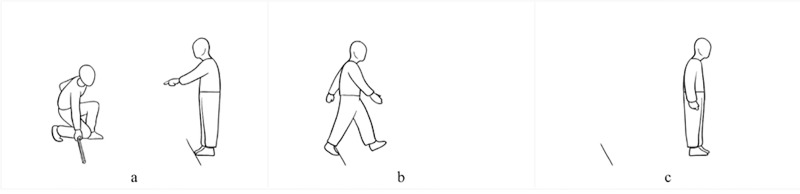
Estimation of the stepping-forward task. One direction (a) and the real performance of the stepping-forward task in the opposite direction (b, c).

First, for the estimated stepping-forward measure, the participant is placed behind a line and is instructed to predict his/her maximum distance for stepping forward (Fig 1). Once the participant indicates he/she understood the procedure, the estimation is collected. For this, the participant stays behind the takeoff line, which is clearly marked on the floor, while the rater, starting at the feet of the participant, slowly and steadily moves a thin wooden stick marker until the participant tells him or her to stop, indicating the maximum estimated distance for stepping forward. Fine adjustments are allowed after the participant gives the order to stop. The estimated measure corresponds to the distance between the line and the wooden stick marker (cm). Second, for the real stepping-forward measure, the participant turns in the opposite direction, staying behind the line (standing in an upright start position with feet slightly apart, head straight and forward, and arms down by the sides of the body) and is instructed to step forward as far as possible, so that both feet pass the takeoff line. The real stepping-forward measure corresponds to the distance between the takeoff line and the foot that is farthest back (cm).

To avoid the learning bias effect between measurements, each participant is tested individually, performing the trial and scoring with a minimum of a 5-min rest break, during which the starting reference line location is changed between trial and scoring attempts.

A test-retest reliability was performed, and intra- and interrater reliability was determined. For the reliability evaluation, a test–retest design was performed in a controlled environment by two fixed raters. The instructions, measuring instruments and test conditions were standardized in order to minimize measurement errors. Each rater measured the same participant twice for the intrarater reliability procedure with a week-long interval between measures. Interrater reliability assessment was performed by two raters, measuring the same participant twice, alternating the instruction randomly. Finally, the construct validity of SF-APT to predict fall occurrence was assessed considering the trial and scoring attempts.

### Data collection

Participants were assessed individually by two trained raters. Participants and raters were blinded to the study’s objectives.

#### Perceptual and stepping-forward boundary

The following outcomes were computed from the distances collected by SF-APT regarding each participant: estimated stepping-forward distance (cm), real stepping-forward distance (cm), algebraic error (difference between real and estimated distances), absolute error (|algebraic error|), absolute percent error (|1–estimated/real performance| x 100), and error tendency frequencies concerning algebraic error (overestimation: real < estimated; underestimation: real > estimated) [22]. These variables measure the error or bias magnitude. Error tendency indicates the error direction, that is, if the bias is under- or overestimated.

#### Falls

Falls were defined as “an unexpected event in which the participants come to rest on the ground, floor, or lower level” [23]. Falls resulting from risky and dangerous circumstances or traffic accidents were not considered. Therefore, only falls occurring during common daily life movements or activities were considered. Fall occurrence in the previous 12 months and the circumstances surrounding each fall were assessed by a questionnaire filled by the evaluator in the form of an interview. A nonfaller was defined as a subject who had not fallen in the previous 12 months, a faller as a subject who had fallen at least once in this period, and a recurrent faller as a subject who had fallen more than once in the same period [2, 3].

#### Complementary measures

Sociodemographic characteristics were assessed by a questionnaire filled by the interviewer. Body composition was evaluated by using a stadiometer (Seca 770, Hamburg, Germany) and an electronic scale (Seca Bella 840) to compute body mass index (m/kg^2^) and by bioimpedance (Omron BF 511, USA) to evaluate body fat and lean mass [24].

### Data analysis

Statistical analyses were performed using the SPSS package version 24 (SPSS Inc., Chicago, IL, USA) and Microsoft Excel (version 16.9, Redmond, USA). Statistical significance was set to *p <* 0.05.

#### SF-APT reliability (inter and intra agreement)

The intraclass correlation (ICC) was used for assessing reliability [25]. In this study, repeated measures analysis of variance (ANOVA) was performed for fixed raters (ICC_2,k_) to evaluate intra- and interrater relative reliability, while the standard error of measurement (SEM) [26] and coefficient of variation (CV) were used to assess the absolute reliability of each parameter [27, 28]. Systematic bias was verified by the F-ratio (with true value 0). The ICC estimates (α—level = 0.05) were calculated using SPSS software, based on a mean-rating (k = 2), absolute agreement, 2-way random average model. Microsoft Excel was used for SEM, CV and F-ratio calculation.

#### SF-APT data exploratory analysis

Descriptive statistics were used to characterize SF-APT participants’ data on absolute error, algebraic error, absolute percent error (mean and standard deviation) and on error tendency (over- and underestimation frequencies). Comparisons between the trial and scoring attempts and between estimated and real stepping-forward distance were performed by a paired sample t test. Normality was assumed based on the central limit theorem for these quantitative variables. Qualitative variables comparisons between trial and scoring attempts were performed using the McNemar Test [29].

An exploratory analysis using univariate binary logistic regressions was performed in order to explore the risk for fall occurrence associated with every single variable accessed by the test. Data are presented as odds ratios (ORs) and 95% CI.

#### Construct validity

The multivariate binary logistic regression analysis and receiver operating characteristic (ROC) analysis were used to select key variables from the SF-APT, which should be included in the fall risk assessment tool, as well as to test the need for a trial prior to the scoring test.

The analyses were performed considering fallers vs nonfallers and recurrent-fallers vs nonfallers for both trial and scoring attempt data. A similar methodology was used by Pereira et al. [30]. First, the fittest multivariate binary logistic regression model was determined by using a traditional approach. For this, all variables that yielded a *p* value < 0.20 in the univariate analysis were candidates for the multivariable model. A model containing all the variables of reported importance was created. Variables that did not meet a significance of *p* < 0.05 in the Wald test were eliminated, and a new model was built. Therefore, the most parsimonious model was built by using the Wald statistic to test the significance of each variable added to the model, and the likelihood ratio was used to compare each new model with the previous model without the variable. The assumption of linearity in continuous variables was checked using the logit function. Outliers and influential points were identified. The overall fit was evaluated using the Hosmer-Lemeshow goodness-of-fit test; a nonsignificant result in the test means a good goodness-of-fit. Second, ROC analysis, based on the area under the curve (AUC), was used to examine the ability of the build models to discriminate fallers from nonfallers and recurrent-fallers from nonfallers.

## Results

The SF-APT was well tolerated since all the participants were able to perform the test correctly, and no adverse events were reported. Moreover, the test accurately assessed the community-dwelling older adults’ perceptions of affordances.

### SF-APT reliability (inter- and intrarater agreement)

The results concerning reliability are shown in Table 1. Intrarater reliability results for SF-APT outcomes were as follows: ICC_2,k_ = 0.95; SEM = 2.99 cm for estimated stepping forward; ICC_2,k_ = 0.97; SEM = 2.70 cm for real stepping forward; ICC_2,k_ = 0.93; SEM = 2.53 cm for algebraic error; ICC_2,k_ = 0.89; SEM = 2.18 cm for absolute error and ICC_2,k_ = 0.83; SEM = 4.28% for absolute percent error. Interrater correlations ranged between ICC_2,k_ = 0.99 for estimated stepping forward, algebraic error, absolute error and absolute percent error and ICC_2,k_ = 1.00 for real stepping forward. The SEM results between raters were 0.49 cm for estimated stepping forward, 0.00 cm for real stepping forward, 0.43 cm for algebraic error, 0.33 cm for absolute error and 0.39% for absolute percent error. No systematic bias was detected with the F test (Table 1).

**Table 1:** Relative and absolute intra- (N = 30) and interrater (N = 34) reliability for the SF-APT outcomes.

Outcomes		Mean ± SD	Relative reliability	Absolute reliability		*F* test	
			ICC_2.k_	SEM	CV	*F*	*p*
Intrarater							
ESF (cm)	test	58.2 ± 12.3	0.95	±2.99	±4.57	0.81	0.72
	retest	60.2 ±13.6					
**RSF (cm)**	test	64.9 ±15.3	0.97	±2.70	±4.09	0.83	0.69
	retest	66.9 ±16.9					
**AlE (cm)**	test	4.3 ±9.6	0.93	±2.53	±0.35	0.95	0.56
	retest	2.5 ±9.9					
**AE (cm)**	test	7.8 ±6.9	0.89	±2.18	±1.18	1.23	0.29
	retest	7.9 ±6.3					
**APE (%)**	test	12.1 ±11.3	0.83	±4.28	±1.17	1.54	0.13
	retest	12.0 ±9.1					
Interrater							
**ESF (cm)**	rater 1	47.1 ±11.0	0.99	±0.49	±4.26	0.99	0.51
	rater 2	46.9 ±11.1					
**RSF (cm)**	rater 1	57.1 ±17.3	1.00	±0.00	±3.29	0.99	0.50
	rater 2	57.1 ±17.4					
**AlE (cm)**	rater 1	10.1 ±13.6	0.99	±0.43	±0.75	1.01	0.48
	rater 2	10.2 ±13.5					
**AE (cm)**	rater 1	13.2 ±10.5	0.99	±0.33	±1.26	1.00	0.50
	rater 2	13.2 ±10.5					
**APE (%)**	rater 1	21.8 ±12.3	0.99	±0.39	±1.77	0.99	0.50
	rater 2	21.9 ±12.3					

SD standard deviation, ICC intraclass correlation coefficient, SEM standard error of measurement, CV coefficient of variation, ESF estimated step forward, RSF real step forward, AlE algebraic error, AE absolute error, APE absolute percent error.

### SF-APT data exploratory analysis

The SF-APT variables on the participants’ results are shown in Table 2. In general, the estimated maximum distance for stepping forward was less than the performed action (underestimation tendency) (algebraic error: trial attempt 4.7 ± 9.8 cm; scoring attempt 6.0 ± 8.5 cm, *p <* 0.001), which is confirmed by the prevalence of an underestimation bias (error tendency: trial attempt 68.0%; scoring attempt 77.2%). However, with other participants, the opposite occurred. These participants showed an overestimation bias (error tendency: trial attempt 32.0%; scoring attempt 22.8%), that is, they estimated a greater distance than what was actually performed (see the algebraic error standard deviations, which are greater than the average value).

**Table 2:** Descriptive statistics for the SF-APT variables (N = 347).

Variables	Trial attempt	Scoring attempt
Estimated stepping-forward (cm)	59.7 ± 15.0*,**	60.9 ± 15.5
Real stepping-forward (cm)	64.4 ± 15.9*,**	66.9 ± 15.4
Algebraic error^†^ (cm)	4.7 ± 9.8*	6.0 ± 8.5
Absolute error (cm)	7.5 ± 7.8	7.7 ± 7.0
Absolute percent error (%)	11.6 ± 11.2	11.5 ± 9.8
Error tendency (%)		
Overestimation	32.0*	22.8
Underestimation	68.0	77.2

^†^[Real-Estimated].

*Significant difference between trial and scoring attempt, *p <* 0.05.

**Significant difference between the estimated and real step forward, *p <* 0.05.

The data are expressed as the mean and standard deviation (± SD) or prevalence in percentage (%).

The absolute and percent errors results showed that participants have a lack of accuracy in estimating distance for the stepping-forward task on the trial and scoring attempts of approximately 7.5 cm and 11.5%, respectively. Comparisons between trial and scoring attempts showed that in the trial attempt, the estimated and the real stepping-forward distance results were smaller (estimated less ~ 1.2 and real less ~ 2.5 cm), as was the algebraic error (less ~ 1.3 cm), *p <* 0.05. Moreover, the underestimation bias increased from the trial to the scoring attempt to 9.2%, *p <* 0.05.

**Fig 2:**
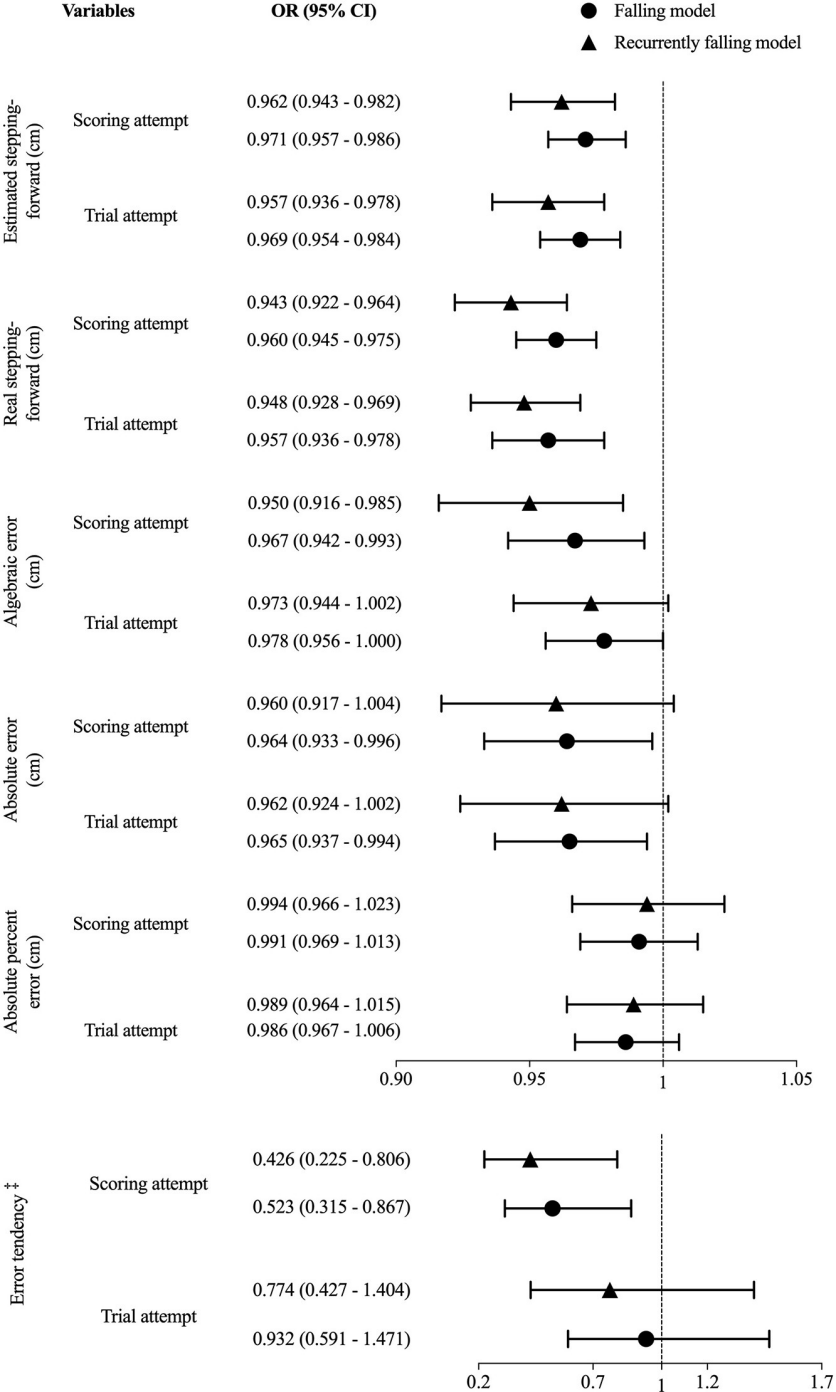
Odds ratio (OR) and 95% confidence interval (CI) of SF-APT variables for the univariate risk of being a faller (N = 347) and of being a recurrent faller (N = 263). ^‡^Underestimation vs overestimation.

The univariate binary regression analysis presented in Fig 2 shows three variables explaining fall occurrence at the trial attempt (OR ranging from 0.957 for real stepping forward to 0.969 for estimated stepping forward), and two variables explaining recurrent fall occurrence (OR of 0.957 for estimated stepping forward and 0.948 for real stepping forward), *p <* 0.05. In the scoring attempt, five variables explained fall occurrence (OR ranging from 0.523 for underestimation ET to 0.971 for estimated stepping forward) and four variables explained recurrent fall occurrence (OR ranging from 0.426 for underestimation bias to 0.994 for absolute percent error). Thus, a higher value in all these variables decreased the likelihood of being a faller or a recurrent faller, as well as the error tendency of underestimation bias (Fig 2).

### SF-APT construct validity

Multivariate binary regression analysis (Table 3) selected the variables estimated step forward and absolute error in interaction with error tendency as the key variables from the SF-APT, which should be included on the fall risk assessment tool, *p <* 0.05. Note that these results only refer to scoring attempt data. In fact, there were no significant results from multivariate regression analysis for the trial attempt data, and it was not possible to build any model with these data.

**Table 3:** Selection of the variables used to access the risk of being a faller and of being a recurrent faller based on multivariate binary logistic regression modeling (Falling vs. Nonfalling Model: N = 347; Recurrent falling vs. Nonfalling Model: N = 263).

Model	Key variables	OR (95% CI)	Model AUC (95% CI)
**Falling**	Scoring attempt for the estimated stepping-forward (cm)	0.964 (0.948–0.979)	0.665 (0.608–0.723)
	Scoring attempt absolute error (cm)^†^ and error tendency^‡^		
	Overestimation		
	Underestimation	0.941 (0.910–0.973)	
Falling recurrently	Scoring attempt estimated stepping-forward (cm)	0.951 (0.931–0.973)	0.728 (0.655–0.797)
	Scoring attempt absolute error (cm)^†^ and error tendency^‡^		
	Overestimation		
	Underestimation	0.914 (0.868–0.962)	

*Interaction between variables.

^†^[Real-Estimated].

^‡^Overestimation as reverence.

Data are multivariate odds ratios (ORs) and 95% confidence intervals (CIs), cut-off points for π, specificity, sensibility, and area under the ROC curve (AUC) and 95% CI.

The Hosmer-Lemeshow goodness-of-fit test was not significant regarding either the falling model (*p =* 0.591) or the recurrently falling model (*p =* 0.241). These two most fit models showed that, for each additional cm on the estimated stepping-forward distance variable, the likelihood of being a faller decreased by 3.6%, OR: 0.964 (95% CI: 0.948–0.979), and the likelihood of being a recurrent faller decreased by 4.9%, OR: 0.951 (95% CI: 0.931–0.973). The modeling results also showed that when the tendency was underestimated, for each additional cm on absolute error, the likelihood of being a faller decreased by 5.9%, OR: 0.941 (95% CI: 0.910–0.973), and the likelihood of being a recurrent faller decreased by 8.6%, OR: 0.914 (95% CI: 0.868–0.962). The falling model revealed an AUC of 0.665 (95% CI: 0.608–0.723), and the recurrently falling model revealed an AUC of 0.728 (95% CI: 0.655–0.797).

## Discussion

The objective of the present study was to develop and assess the validity and reliability of the SF-APT for fall risk assessment in community-dwelling older adults. Based on our results, in the literature review and feedback from the expert reviewers, the SF-APT was shown to be a valid and reliable tool to assess fall risk in community-dwelling older adults. Reliability tests indicated excellent correlations and small standard errors between measurements [31] for both intrarater and interrater analyses. The criterion validity could not be established due to the lack of a similar assessment tool. As an indication of construct validity, the SF-APT was able to significantly discriminate individuals who were regular fallers and those who were occasional fallers, namely, at the scoring attempt.

These results confirmed the expert opinions and participant feedback that there is a need for a trial in the assessment protocol in order to ensure that participants could understand the administration procedures. Therefore, the trial ensures that participants are able to estimate and perform their maximum stepping-forward distance at the scoring attempt. In addition, the experts’ observation of the test application confirmed that the estimation and action boundary should be measured in different places to avoid the presence of any allocentric frame of reference. Single SF-APT outcomes were shown to significantly explain fall occurrence; however, the estimated stepping forward and absolute error in interaction with error tendency were selected as the key outcomes to explain this negative event. The results showed that a large stepping-forward estimation associated with an underestimation bias works as a protective mechanism for falling and recurrent falling.

Our findings complement the results of previous studies regarding the perception of affordances in young and older adults [15, 18, 32]. Noel and colleagues found that older adults perform an overestimation judgment error of 11 cm in the stepping over an obstacle task compared to young adults [15]. Furthermore, the studies of Konczak et al. and Cesari et al. in the stair climbing task concluded that older adults could perceive their actual stair capability as well as young adults could, despite the change in the action capability with aging [18, 32]. Nevertheless, it should be noted that the studies mentioned above examined the perception of action boundary but did not examine fall occurrence. Moreover, our findings are in accordance with those of Noel and colleagues [15], who hypothesized that an overestimation bias on stepping over an obstacle could be a risk for falls by showing that, in opposition to overestimation bias, the underestimation bias decreases the likelihood of falls. Thus, within the framework of the ecological approach, falls can be regarded as failed actions that result from inaccurate affordance perception, our study results indicate that falls may occur due to an overestimation mismatch between what the older adults believe they are able to do and what they are actually capable of doing. Such a discrepancy could lead to older adults endangering themselves by performing actions that they are no longer physically capable of performing.

Therefore, SF-APT was shown to address key components useable for the assessment of fall risk. The calculated AUCs for fallers and for recurrent fallers discrimination were low/moderate, suggesting that the test complements other methods for fall risk assessment, such as balance or gait tests. For example, the Fullerton Advanced Balance (FAB) scale, which showed an overall perdition success rate of 71.4% [33]. This would consider the multifactor nature of fall occurrence (intrinsic vs extrinsic factors, plus accidental, or exposure over time) based on Palumbo et al. and Klenk et al. [7, 9] and therefore address the causes of falls that the affordance´s perception assessment does not address.

Considering the results, we believe that for fall risk assessment, it would be relevant to address other locomotor tasks, such as stepping to the side, stepping up onto a platform or stepping over an obstacle, instead of one single task. In fact, Kuft et al. [34] observed that the task of stepping over a raised bar best integrated the criteria for the affordance construct with regard to perceived and actual physical ability, particularly for stepping. In addition, the reliability tests could be assessed in real-life environments/situations because the secure environment provided may not be generalizable risky real-life situations. A limitation of the present study was that falls were assessed retrospectively; nonetheless, we observed that similar methodology was used to validate several fall risk assessment instruments, such as the BERG scale [35] and, more recently, the FAB scale [33, 36]. Future research focusing on this subject should address prospective falls in order to improve construct validity accuracy and involve populations with cognitive impairments or institutionalized older adults. Moreover, it would be of interest to investigate the associations between SF-APT outcomes and fear of falling or balance, for instance. Reference values for both men and women regarding SF-APT outcomes and respective cut-offs to discriminate fallers from nonfallers should be investigated.

Finally, participants and raters revealed good acceptance of the SF-APT, considering it as a quick (10–15 min), easy and inexpensive way to assess the ability and accuracy to perceive action boundary for stepping-forward in community-dwelling older adults. Moreover, the material used is widely available and easy to transport. The SF-APT was well tolerated since all the participants were able to perform the test correctly, and no adverse events were reported. This new field test might complement the easy test [37] and relevant batteries for functional assessment in older adults [33, 38–40], adding a specific exam to evaluate the perception of affordances and potentially increasing their ability to discriminate the older adults who are at risk for falling, despite being based on retrospective fall occurrence. SF-APT assesses the ability and accuracy to perceive action boundaries, filling a gap that the determinant factors addressed in previous studies failed to explain. Moreover, the test outcomes matched the test aims.

## Conclusions

SF-APT accurately measured the perceptual and stepping-forward boundary, quantifying the accuracy bias and proving to be a reliable and valid method for fall risk assessment in community-dwelling older adults.

There must be a trial prior to the test scoring. Selected fall risk assessment key outcomes showed that a large estimated stepping forward associated with an underestimated absolute error works as a protective mechanism for falling and recurrently falling.

SF-APT is safe, quick, easy to administer, well accepted and reproducible for application for community-dwelling older adults in community or clinical settings by either clinical or nonclinical care professionals.

## Supporting information

S1 File The Stepping–forward affordance perception test (SF-APT). (DOCX)
