# Supplementary material for: Reliability and construct validity of the stepping-forward affordance perception test for fall risk assessment in community-dwelling older adults
Source: PLoS One. 2019 Nov 20;14(11):e0225118. doi: 10.1371/journal.pone.0225118 (PMC6867623; doi:10.1371/journal.pone.0225118)
Supplement: S1 File — (DOCX) [file pone.0225118.s001.docx]

**The Stepping – forward *affordance* perception test (SF-APT)**

*SF-APT performance involves a first training attempt (trial) and a second measurable attempt (scoring). The trial and the scoring measures are performed with a minimum of 5 minutes rest break. Both test trial and scoring tasks are performed individually on a uniform floor, but in different locations. The test begins with the rater providing a verbal explanation followed by the trial, with no feedback or record. Record the estimated stepping-forward measure and the real stepping-forward measure.*

**Purpose**

Quantifying first perceived and second real stepping-forward ability.

**Material required**

Thin wooden stick; Tape measure to measure distance estimated and stepped; Non-slip and uniform floor.

**Pre-test explanation**

Explain the test procedures to the participant. Perform screening of health conditions and risks and obtain informed consent. Prepare forms and register basic information such as birth date, gender and other important information. The takeoff line should be clearly marked.

*Estimated stepping-forward measure*

**Procedure**

The participant is placed behind the takeoff line and is instructed to predict his/her maximum distance for stepping-forward. The rater slowly and steadily moved a thin wooden stick marker until the participant tell to stop, indicating the maximum estimated distance of stepping-forward. The participant was given opportunity to adjust the distance.

**Scoring**

The estimated measure corresponds to the distance between the takeoff line and the wooden stick marker.

*Real stepping-forward measure*

**Procedure**

The participant turns to the opposite direction, staying behind the takeoff line (standing in an upright start position with feet slightly apart, head straight and forward, and arms down by the sides of the body) and is instructed for step forward as far as possible, so that both feet pass the takeoff line, one at a time.

**Scoring**

The measurement is taken from takeoff line to the most backward foot.

| **Test Date**: | **Participant Gender** | **Participant Birth Date** | **Examiner ID**: |
| --- | --- | --- | --- |
| ____/___/___  yyyy/ mm/ dd | M \| F | ____/___/___  yyyy/ mm/ dd | **_____________________________________** |

| **Participant ID** | **Estimated stepping-forward (cm)** | **Real stepping-forward (cm)** | **Observations** |
| --- | --- | --- | --- |
| **#1** |  |  |  |
| **#2** |  |  |  |
| **#3** |  |  |  |
| **#4** |  |  |  |
| **#5** |  |  |  |
| **#6** |  |  |  |
| **#7** |  |  |  |
| **#8** |  |  |  |
| **#9** |  |  |  |
| **#10** |  |  |  |
| **#11** |  |  |  |
| **#12** |  |  |  |
| **#13** |  |  |  |
| **#14** |  |  |  |
| **#15** |  |  |  |
| **#16** |  |  |  |
| **#17** |  |  |  |
| **#18** |  |  |  |
| **#19** |  |  |  |
| **#20** |  |  |  |
